# Alteration of acute toxicity of inorganic and methyl mercury to *Daphnia magna* by dietary addition

**DOI:** 10.1038/s41598-021-02300-4

**Published:** 2021-11-24

**Authors:** Christopher A. Hylton, Martin Tsz-Ki Tsui

**Affiliations:** 1 Department of Biology, University of North Carolina at Greensboro, Greensboro, NC 27402 USA; 2 School of Life Sciences, The Chinese University of Hong Kong, Shatin, N.T., Hong Kong SAR China

**Keywords:** Biogeochemistry, Environmental sciences

## Abstract

Acute toxicity of inorganic mercury [Hg(II)] and methylmercury (MeHg) to *Daphnia magna* was characterized using a 48-h static, non-renewal acute toxicity test, in which we compared the toxicity of Hg(II) and MeHg in the absence (water-only) and presence of diet [green alga (*Raphidocelis subcapitata*), yeast, Cerophyll, and trout chow (YCT), or both]. Overall, Hg(II) is more toxic to *D. magna* than MeHg, with 48-h median lethal concentrations (LC50s) being 4.3 µg/L (95% confidence interval: 4.1–4.5 µg/L) for Hg(II) and 14.3 µg/L (13.2–15.3 µg/L) for MeHg. For Hg(II), the addition of any diet would significantly increase its 48-h LC50, but the 48-h LC50 for MeHg decreased significantly to 7.1 µg/L (6.4–7.8 µg/L) with the algal addition. We also show that the addition of diets significantly influenced the levels and speciation (dissolved vs. particulate) of both Hg forms in the test solution. The bioaccumulation of Hg(II) and MeHg was impacted by the dietary addition, and it appears that the body residue level triggering mortality varied widely among treatments. The results suggest that standard short-term toxicity tests (water-only) should be supplemented with extra tests with dietary addition to provide a more environmentally relevant estimation of short-term toxicity of chemical compounds.

## Introduction

One of the major goals of ecotoxicology is to determine a “safe” level of environmental chemicals for protecting the health of wildlife in the environment. Bioassays, or toxicity tests, were thus developed as a tool to assess at what chemical concentrations and exposure duration we would be able to observe harmful effects of the chemicals^1^. In general, toxicity tests are divided into short-term, acute tests and long-term, chronic tests. Among many studies, the acute toxicity test is still the more popular approach in examining chemical toxicity mainly because of its simplicity, short duration and an easily recognizable endpoint, even though we all recognize that the chronic test examines toxicity endpoints (e.g., growth and reproduction) and chemical levels with higher relevance to the environment^1^. In aquatic toxicology, toxicity testing with *Daphnia* spp. represents about half of all studies examining the toxic effects on aquatic invertebrates^2,3^, in which lethality or immobilization is the most common endpoint^4,5^. It should be noted that recent interests in other physiological acute endpoints such as feeding activity and heart rate in *Daphnia* spp. exist^6^.

Standard toxicological tests with *Daphnia* spp. (either *D. pulex* or *D. magna*) most often expose the neonates (< 24 h old) to a chemical pre-dissolved at multiple concentrations in the dilution water for a total of 48 h (i.e., static and non-renewal), and determine the 48-h median lethal concentrations (LC50s) of the chemical to this standard organism^5^. Almost all of the earlier studies examined waterborne chemical toxicity to *Daphnia* spp.^7^ and/or examined how the water chemistry can influence the toxicity and bioavilability of waterborne chemicals such as heavy metals^8^, which led to the ultimate development of Biotic Ligand Model in early 2000s^9,10^. In the last two decades, there has been increasing evidence that dietborne metal toxicity is more ecologically relevant and the effects can be quite different from those of aqueous metal exposure^11–13^. However, research investigating dietborne toxicity mainly focused on the long-term, chronic toxicity with *Daphnia* spp. or other freshwater and marine organisms, with growth and reproduction as the most common endpoints^11,14–17^.

Dietary toxicity of metals to aquatic organisms is more complicated than aqueous exposure due to the additional consideration for the partitioning of metals between water and diet and the eventual metal uptake by the test organisms^12,18^. Interestingly, dietborne metal concentrations may not be the best predictor of its toxicity to the testing animals because ingestion/feeding rate and bioavailability (i.e., assimilation of dietborne metals) may be negatively affected at higher concentrations of dietborne metal exposure due to the toxic nature of metals^19^. This possibility is certainly in a stark contrast to the scenario of aqueous exposure alone since dissolved metal concentration is often a very strong predictor of animal mortality in short-term toxicity tests^20–22^.

However, little is known about how the presence of diet(s) may alter the acute toxicity of metals to a standard test organism during short-term exposure. In fact, the standard chronic toxicity assays with the standard test organisms (e.g., 21-d test with *Daphnia* spp.) by including diet (usually green algae and/or other supplements) would allow the organismal exposure to chemicals through both dissolved and dietary routes^15,23,24^. Under these conditions, dissolved metals may partition to food material thus providing for a dietary exposure route^12,25^.

In this study, we examined whether the addition of standard diets (e.g., green alga and yeast, Cerophyll, and trout chow, or YCT), which would be typically administrated in standard chronic reproduction tests with *Daphnia* spp.^23,26^, would alter the acute toxicity of two mercury (Hg) compounds (inorganic mercury(II) and methylmercury) to *Daphnia magna*. In order to offer further insights into the partitioning of Hg between diets and water, we analyzed concentrations of both Hg compounds in unfiltered and filtered exposure media at the end of the bioassays to distinguish these two phases of Hg under the influence of the different dietary types. At the end of each bioassay, we collected the mobile daphnids and measured the biota Hg levels under different exposure concentrations and dietary conditions.

Due to their differing dietary uptake rates^24^, we first hypothesized that the addition of alga would enhance the toxicity of MeHg compared to its unamended control while it would decrease the toxicity of Hg(II) compared to its unamended control. Further, we hypothesized that the addition of different dietary types (i.e., alga and YCT) would produce the same effect on the toxicity of both Hg compounds as compared to their respective unamended controls due to the quick binding of Hg(II) and MeHg to the diets^24^. Our work demonstrates that the presence of different diets changes the 48-LC50s of both Hg compounds to *D. magna*, and interestingly we show that the Hg bioaccumulation has been considerably altered by the presence of diets, substantially enhancing the body residue levels of Hg required to cause 50% mortality.

## Results and discussion

### Variations of 48-LC50s under different dietary addition regimes

There was a range of 48-h LC50s for Hg(II) and MeHg to *D. magna* under different dietary addition regimes (i.e., no food, + alga, + YCT, and + alga & YCT), through calculations based on the measured initial concentrations [either as Hg(II) or MeHg], their average measured unfiltered concentrations (at t = 0 h and t = 48 h), and their average measured filtered concentrations (at t = 0 h and t = 48 h) (Table 1).

**Table 1:** Calculated 48-h median lethal concentration (LC50) and the associated 95% confidence interval for inorganic mercury [Hg(II)] and methylmercury (MeHg) to *Daphnia magna* under different treatments.

Treatment	Hg(II) based on initial Hg only	Hg(II) based on average of unfiltered Hg	Hg(II) based on average of filtered Hg
**48-h LC50 (95% confidence interval) (µg/L)**			
No food	5.15 (4.88–5.43)	4.32 (4.11–4.54)	4.16 (3.96–4.37)
+ Alga	10.34 (9.43–11.35)	8.98 (8.30–9.71)	6.94 (6.36–7.57)
+ YCT	26.35 (25.51–27.22)	17.63 (17.09–18.18)	14.22 (13.77–14.68)
+ Alga & YCT	28.10 (27.45–28.77)	18.57 (18.14–19.00)	14.97 (14.61–15.33)

Treatment	MeHg based on initial Hg only	MeHg based on average of unfiltered Hg	MeHg based on average of filtered Hg
No food	14.63 (13.64–15.68)	14.33(13.35–15.39)	14.07 (13.12–15.09)
+ Alga	7.70 (7.01–8.45)	7.08 (6.40–7.84)	5.59 (5.01–6.23)
+ YCT	19.16 (18.01–20.38)	14.23 (13.24–15.29)	10.93 (10.13–11.79)
+ Alga & YCT	21.19 (19.37–23.18)	16.11 (14.54–17.86)	12.13 (10.96–13.43)

Three values of 48-h LC50 values of Hg(II) and MeHg are based on measured initial concentrations only (*column 1*), average of initial and final unfiltered concentrations (*column 2*), or average of initial and final filtered concentrations (*column 3*).

For Hg(II), regardless of the calculation methods, 48-h LC50s were the lowest for the treatment without food addition, and was significantly lower (*p* < 0.05) than the treatment of algal addition. For both treatments with YCT and Alga & YCT, their 48-h LC50s were significantly higher (*p* < 0.05) than the treatment with algal addition alone. The results indicated that YCT dominated the effects over alga on Hg partitioning to solid particles, which corroborates with the much higher amount of YCT than algal cells (18 µg/mL vs. 10^5^ cells/mL).

For MeHg, regardless of calculation methods, 48-h LC50s were the lowest for the treatment with algal addition, being significantly lower (*p* < 0.05) than all other treatments. For calculation based on initial Hg only (i.e., *column 1* in Table 1), the treatment without food had significantly lower 48-LC50 than both treatments with YCT and Alga & YCT. However, such significant differences in 48-LC50s disappeared if the calculation was based on average unfiltered Hg (i.e., *column 2* in Table 1). If the calculation was based on average filtered Hg (i.e., *column 3* in Table 1), the treatment with YCT addition had no significant difference (*p* > 0.05) in 48-h LC50 with the treatment with Alga & YCT addition, but had significantly lower 48-h LC50 (*p* < 0.05) than the treatment without food.

These results demonstrated that food addition can significantly alter 48-h LC50s of both Hg forms to *D. magna* and the specific approach we used to calculate LC50 values could affect the outcome of the treatment comparison. Since Hg is known to bind to the diets employed in this study (i.e., YCT and algae^24,27^), we decided to use average unfiltered Hg values for all 48-h LC50 calculations (i.e., *column 2* in Table 1) for further discussions throughout this study because the exposed organisms can take up Hg in both dissolved and dietary fractions^24^.

### Temporal changes of Hg levels and speciation in the exposure media

**Figure 1:**
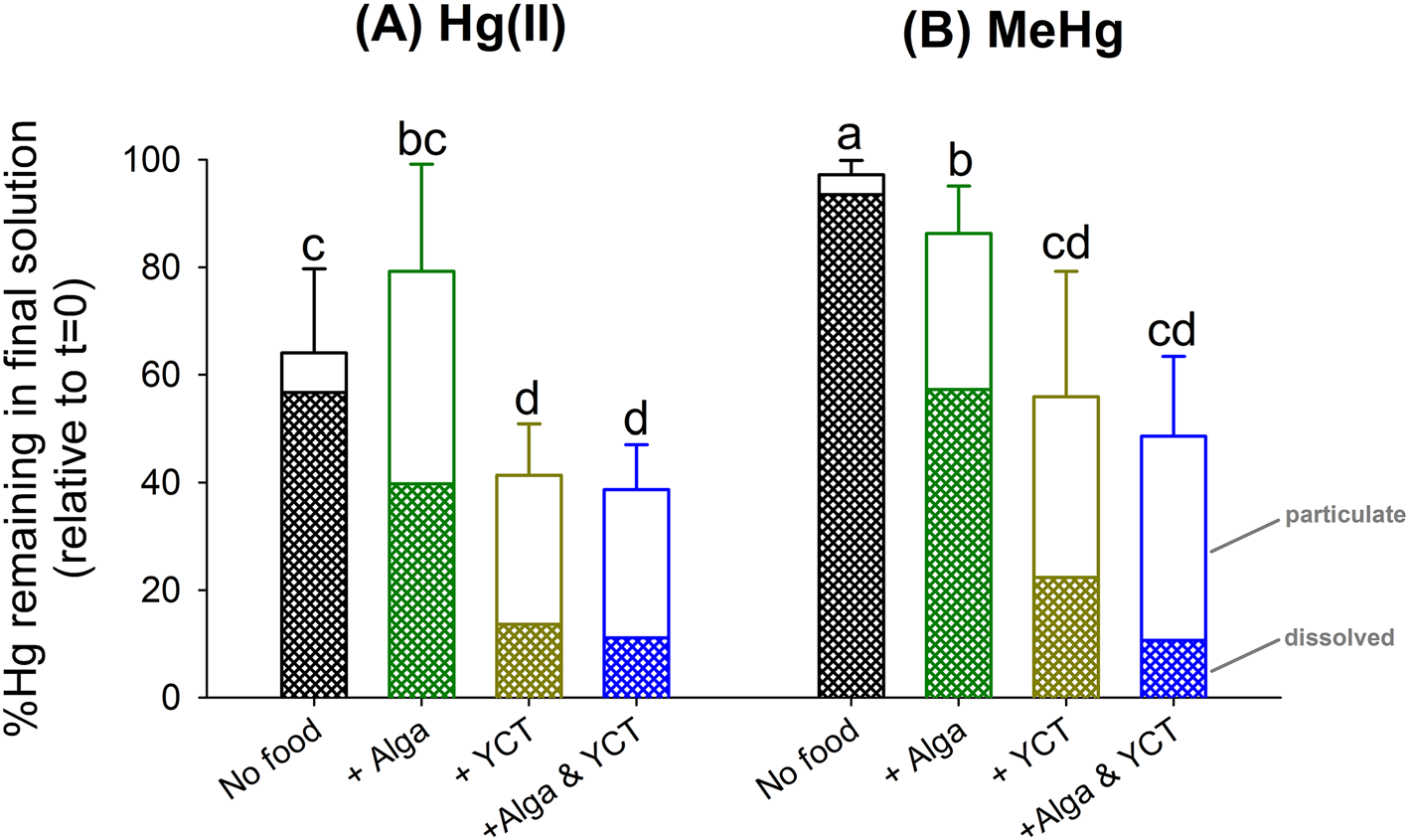
Bar graphs showing the percentage of mercury (Hg) remaining in the solution (relative to initial (t = 0) value) and the percentage as dissolved vs. particulate form after 48 h of exposure under different treatments (No food; + Alga; + YCT; + Alga & YCT) for (**A**) inorganic Hg [Hg(II)] and (**B**) methylmercury (MeHg). Means for a treatment are not significantly different (*p* > 0.05) if they bear the same letter. Error bars are standard deviations of average values (n = 5) per treatment. *See* original concentration data in the supplemental information file.

When we compared the initial unfiltered Hg and the final unfiltered Hg, there were significantly higher levels (*p* < 0.05) of MeHg than Hg(II) remaining in the solution at the end of the toxicity tests without food addition (Fig. 1), the differences became not significant (*p* > 0.05) for all treatments with diet addition. This result indicates the “more reactive” nature of Hg(II) with potential binding sites such as the interior surface of the glass beaker, daphnid uptake, or just the outer carapace of daphnids, etc.

With the addition of diets [except for the treatment of algal addition in Hg(II)], diet significantly reduced remaining amount of Hg (*p* < 0.05) in the solution compared to the treatment without food after 48 h of exposure (Fig. 1). Since we stirred the final solution thoroughly and carefully collected the final solution for Hg analyses, this result would mean actual uptake of Hg by daphnids and/or potentially some diet-borne Hg being aggregated or loosely attached to the container wall (*personal observations*).

Regarding the speciation of Hg in the dissolved vs. particulate phase in the final solution, the vast majority of Hg (i.e., 88% for Hg(II) and 96% for MeHg) was in the dissolved phase without diet addition (Fig. 1). Upon algal addition, a much lower proportion of dissolved Hg(II) (50%) and MeHg (66%) was found in the final solution. With YCT addition, an even lower proportion of dissolved Hg(II) (34%) and MeHg (40%) was found. Finally, with the combined addition of both YCT and alga, we found the lowest proportion of dissolved Hg(II) (18%) and MeHg (21%). Through these analyses, the uptake route of Hg (i.e., dissolved vs. particulate) would be significantly altered upon diet addition in this study.

### Mercury accumulation in mobile daphnids

**Figure 2:**
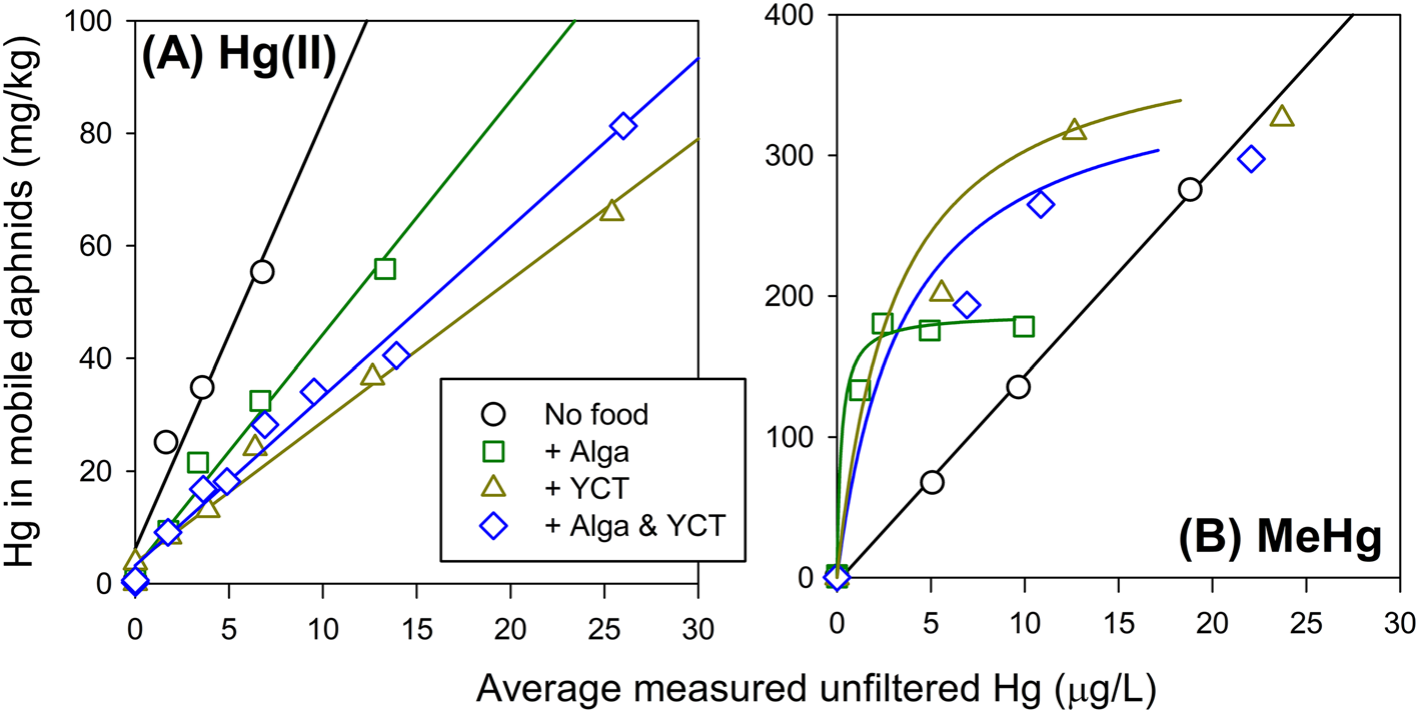
Biota concentrations of (**A**) inorganic Hg [Hg(II)] and (**B**) methylmercury (MeHg) in mobile (surviving) daphnids at the end of the 48-h acute toxicity tests under different treatments: No food; + Alga; + YCT; + Alga & YCT. *See* original data in the supplemental information file.

For Hg(II), the biota Hg levels increased linearly with aqueous Hg(II) concentrations among the four treatments (Fig. 2A). For MeHg, we found that the only treatment showing linear increase of bioaccumulation with aqueous concentration was the control treatment without food addition (Fig. 2B). When comparing with the Hg(II) bioaccumulation with no food, MeHg uptake had a considerably higher value under similar aqueous MeHg exposure, indicating a much higher rate of dissolved uptake of the methylated form of Hg from water that corroborated with previous findings of dissolved uptake tested at much lower Hg exposure^24^.

In all three dietary treatments, the results showed that at lower aqueous concentrations (e.g., ~ 1–10 µg/L) there were much higher MeHg bioaccumulation in the dietary treatments than the control treatment without food (Fig. 2B), while we also observed higher, but to a lesser degree, MeHg bioaccumulation in the dietary treatments than the control treatment without food at higher aqueous concentrations (e.g., > 10–20 µg/L). Apparently, there were “quick” saturation of MeHg uptake at higher concentrations. In fact, the MeHg uptake data from the three dietary treatments followed Michaelis–Menten kinetics, with the saturation values (V_max_) being 188 mg/kg (+ Alga; *r*^2^ = 0.995), being much elevated for treatments with YCT at 396 mg/kg (+ YCT; *r*^2^ = 0.988) and at 367 mg/kg (+ Alga & YCT; *r*^2^ = 0.991).

The very different bioaccumulation patterns between dissolved and dietary uptake of MeHg in this study may be attributed to the very efficient scavenging of MeHg by the diets and efficient assimilation into the biota than Hg(II)^24,28^. Nevertheless, we speculated that the uptake saturation of MeHg may be due to the incomplete digestion of the diets, leading to some of the food-borne MeHg being “purged” out of the gut immediately (i.e., not assimilated into the tissues)^29^.

### Using bioaccumulated mercury to explain daphnid survival

The majority of toxicity studies examined LC50 values based on organismal survival and (nominal or measured) aqueous concentrations, while research on persistent organic pollutants have attempted to determine the critical body burden or median lethal body burden (LBB50) as these biota residual levels would account for the actual amount of toxicants accumulated in the biota and how that would relate to the mortality and other sublethal endpoints^30^.

**Figure 3:**
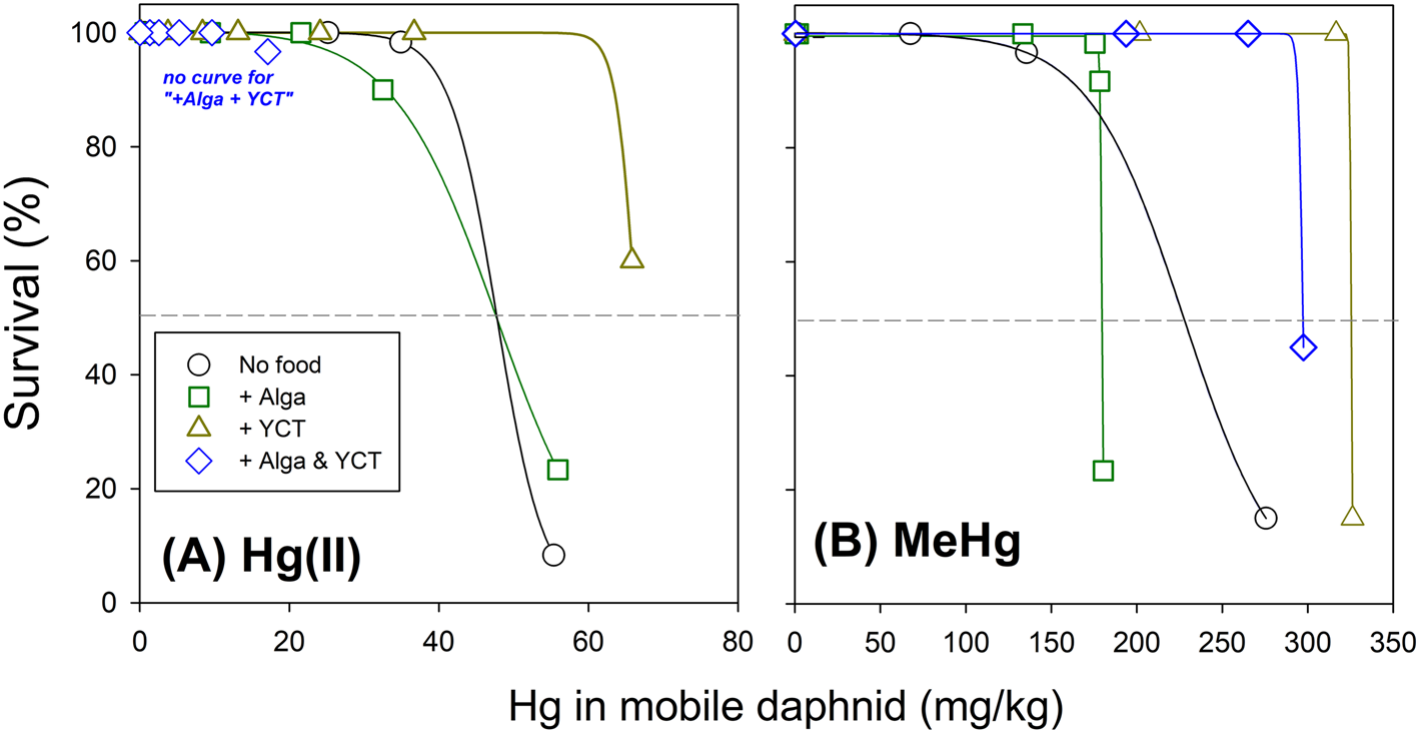
Relationship between biota mercury (Hg) in mobile (surviving) daphnids and the survival rate of the test population for (**A**) inorganic Hg [Hg(II)] and (**B**) methylmercury (MeHg) after 48 h of the acute exposure under different treatments: No food; + Alga; + YCT; + Alga and YCT. Note that the horizontal dash line indicates 50% of survival. In (A), note that no survival curve was plotted for “ + Alga & YCT” treatment.

In this study, we attempted to create the survival curves against biota Hg in mobile (surviving) daphnids under treatments resulting in no or partial mortality, thus excluding the daphnids from treatment levels with 100% mortality. As shown in Fig. 3, even with such limited sample analysis, it could be noted that a considerably higher amount of MeHg was required to elicit 50% mortality than Hg(II). Also, the treatment with or without YCT increased the graphically estimated LBB50 values of both Hg(II) and MeHg (indicated on the graph) compared to the control treatment and treatment with algal addition. For Hg(II), there were almost identical LBB50 values (graphically estimated) for both control treatment and treatment with algal addition.

Therefore, it could be argued that the lower 48-LC50s of Hg(II) (control treatment and algal treatment) and MeHg (algal treatment) were not simply due to enhanced uptake of both Hg forms, rather it would be due to the differences in fully assimilated Hg(II) or MeHg leading to mortality. Due to the short amount of time (i.e., 48 h), we speculated that YCT-associated Hg(II) or MeHg may not be fully assimilated and incorporated into the biota in order to exert toxic effects^24^, thus apparently increasing their estimated LBB50 values (Fig. 3).

### Implications to standard toxicity testing

Standard acute toxicity tests to freshwater and saltwater organisms mainly rely on aqueous phase exposure due to its simplicity, reproducibility, and comparability^4,5^. In the last few decades, different research groups have shown the importance of dietborne bioaccumulation of metals and more importantly on the dietborne metal toxicity, which sparked new interests in dietborne metal toxicity in the field in early 2000s^11,14,15^. While the data appeared to have a large uncertainty whether dietborne metal would be more toxic than aqueous metal, so far the results were mainly based on chronic toxicity endpoint. This current study was initiated to tackle the question about acute toxicity of two common forms of environmental Hg in the aquatic environment in the absence and presence of diets recommended by standard toxicity tests with *Daphnia magna*. Our results show clearly that addition of diet would alter LC50, LBB50 (though not calculated directly), bioaccumulation, and changes in concentrations and speciation in the exposure media of two forms of Hg, i.e., Hg(II) and MeHg. Further, we suggest that studies will be needed to characterize the effects of other common diets (e.g., bacterial diets) and food levels (e.g., different algal concentrations) on the toxicity of Hg (and other metals) to daphnids.

Metals and metalloids such as Hg^24^ and Se^31^ have long been known to contribute to dietborne toxicity while other metals such as Ag and Cu have been realized at a more recent time^13^. For Hg uptake by *Daphnia*, a previous study has demonstrated that environmentally realistic Hg bioaccumulation is mainly governed by the aqueous uptake of Hg(II) and dietary uptake of MeHg^24^. Regardless, the ultimate goals of these assessments are to provide toxicity estimation of these contaminants relevant to the realistic aquatic environment in order to offer sufficient protection to aquatic organisms in nature^4,5,23,26^. Therefore, acute toxicity tests performed with feeding could complement tests performed using standard methods by revealing any potential contributions of dietary exposure of chemicals to animal toxicity.

## Methods

### Animal cultures and food preparations

A stock culture of *Daphnia magna* was purchased from Carolina Biological Supply Company (Burlington, NC, USA), and the animal cultures were maintained in pre-aerated Elendt M7 culture media^4^ under a constant temperature (20 ± 1.0 °C) and photoperiod of 16-h light and 8-h dark in an environmental chamber (Thermo Fisher Scientific, Waltham, MA, USA). The cladocerans were fed with a mixture of green alga (*Raphidocelis subcapitata* or previously known as *Selenastrum capricornutum*) at 10^5^ cell/mL and yeast, Cerophyll, and trout chow (YCT; our stock contained fish flake food, dry yeast, and alfalfa leaves combined at a rate of 5 g of each item per 1 L) at 1.5 mL per 1 L culture three times a week according to USEPA^5^. Prior to each feeding, we renewed half of the culture media with pre-aerated Elendt M7 culture media^20^.

The culture of *S. capricornutum* was obtained from UTEX Culture Collection of Algae (Austin, TX, USA) (culture stock ID: 1648). The alga was grown in WC medium for freshwater phytoplankton^32^ under continuous aeration with filtered ambient air, at room temperature (22–25 °C), and sunlight (set at windowsill) yielding algal culture just reaching stationary phase prior to feeding and use in experiments. Algal cell count was performed with a haemocytometer under a compound microscope. Algal cells were concentrated by centrifuge, resuspended in Elendt M7 culture media, and kept at 4 °C in the dark prior to use, usually being consumed within 2 weeks. Each week, we thawed a previously frozen bottle of YCT (a batch of 50 mL) and kept it at 4 °C for the use during the entire week^5^. Our measurements indicated that our prepared YCT solution had 12–13 mg solids/L.

### Acute toxicity tests

We conducted 48-h acute toxicity tests on < 24 h old nenoates of *D. magna* exposed to a control and a series of increasing concentrations of Hg(II) or MeHg under the following treatments: water-only (No food), addition of *S. capricornutum* at 10^5^ cells/ml (+ Alga), addition of YCT at 1.5 mL/L or ~ 18 mg/L (+ YCT), or a combined addition of both *S. capricornutum* and YCT at the above concentrations (+ Alga & YCT).

We obtained inorganic Hg [Hg(II)] as HgCl_2_ (ACS reagent, ≥ 99.5%) from Sigma-Aldrich (St. Louis, MO, USA) while we purchased methylmercury [MeHg] in the form of aqueous CH_3_HgCl solution (1,000 ppm) from Alfa Aesar (Haverhill, MA , USA). The purity of MeHg in the stock solution was verified by MeHg analysis by cold vapor atomic fluorescence spectrometry with gas chromatography and pyrolysis as described in a previous study in our laboratory^33^. Both Hg forms were individually diluted to 1 µg/mL by nanopure water (at 18.2 MΩ/cm) and stored in glass bottles at 4 °C in the dark prior to use. On the day of acute toxicity tests, we prepared one control and five nominal concentrations of Hg(II) or MeHg (overall range from 1.6 to 160 µg Hg/L) in a 1 L glass media bottle, and 200 mL of the solution at each treatment level was poured into an acid-cleaned 250 mL glass beaker with triplicate per each level (i.e., 6 × 3 = 18 beakers per test). We collected ~ 100 mL of solution from each level to determine the actual initial Hg(II) or MeHg concentrations (at t = 0).

We randomly selected 20 neonates from the stock cultures and an individual organism was transferred with an acid-cleaned plastic Pasteur pipette (with a widened opening) to an intermediate beaker with the test solution before transferring to the actual test beaker (with each neonate in 10 ml of test solution), in order to minimize the transfer of any animal waste and exposure media from the stock cultures to the actual test solution. The acute toxicity tests were conducted for a total of 48 h with beakers covered with a plastic wrap at a constant temperature (20 ± 1.0 °C) and photoperiod (16 h light and 8 h dark).

Upon completion of the acute toxicity test after 48 h, we recorded the number of the test organisms with immobility in each test beaker. After daphnids were scored, we transferred the mobile (surviving) individuals from each beaker to another beaker containing clean culture media and allowed the daphnids to depurate for 10–15 mins^24^. We then collected daphnids on a 0.45-µm acetate cellulose filter membrane and rinsed with deionized water in a disposable filter unit (Nalgene, Thermo Fisher Scientific) to remove any non-accumulated test solution with Hg(II)/MeHg on the carapace. The animals were later frozen and then lyophilized with a bench top freeze dryer (SP Scientific, Warminster, PA, USA), and samples were stored dry prior to Hg analysis (*see below*).

For the control and each treatment level, we combined the solution from the triplicate beakers, filtered half of the solution (~ 300 mL) for determining dissolved Hg concentrations through a disposable filter unit (Nalgene, Thermo Fisher Scientific) with 0.45-µm acetate cellulose filter membrane, and kept the other half of the solution (~ 300 mL) for analyzing unfiltered Hg concentrations (at t = 48 h). All water samples (initial sample, final unfiltered sample, and final filtered sample) were preserved with 5% of bromine monochloride (BrCl) and stored in acid-cleaned glass vials with PTFE-lined septa (Thermo Fisher Scientific) prior to Hg analysis.

### Analytical measurements

All unfiltered (initial and final) and filtered (final only) water samples were fully oxidized with 5% BrCl at 80 °C in a water bath to break down ligands binding to Hg(II) or MeHg and/or convert MeHg to Hg(II). For dry daphnid samples, we carefully placed the daphnids on the filter membrane into a Teflon digestion vessel (Savillex, Eden Prairie, MN, USA) and added 5 mL of reagents (4 mL of trace metal grade HNO_3_ (70% w/v) and 1 mL of reagent grade H_2_O_2_ (30% w/v), both from Thermo Fisher Scientific), and the digestion vessel was wrench-tightened and maintained at 80 °C in a water bath overnight to complete the sample digestion^34^. For all sample digestions, we included a reagent blank and a certified reference material (National Research Council of Canada’s TORT-3 Lobster Hepatopancreas) to ensure quality control. Upon completion of the heating step, we added 100 µL of BrCl to complete the digestion steps. Therefore, for each treatment with Hg(II) or MeHg treatments, we measured only total-Hg concentrations. The reagent blank was found to have Hg at ~ 8 pg/mL while our measured Hg concentrations for TORT-3 averaged at 281.1 ± 12.1 ng/g (*n* = 5) (vs. certified values of 292 ± 22 ng/g).

Prior to total-Hg analysis, we neutralized the excessive BrCl in all samples by the addition of 30% HONH_2_·HCl solution (Alfa Aesar). We added aliquots of the water samples (from 0.1 to 100 mL, depending on the expected Hg levels) and biota digest (from 0.1 to 1.0 mL, depending on the expected Hg levels) into 100 mL nanopure water (at 18.2 MΩ/cm) in a glass bubbler. We then added 0.2 mL of 20% SnCl_2_ solution (Alfa Aesar) to completely reduce Hg(II) to Hg(0), and purged Hg(0) with Hg-free ultrapure N_2_ gas to a pre-blanked gold trap. Gold trap was later heat-desorbed to release sample Hg as Hg(0), and Hg was detected by a Brooks Rand Model III cold vapor atomic fluorescence spectrometry detector (Brooks Rand Instruments, Seattle, WA, USA). All samples were run in duplicate and the relative standard deviation between duplicate measurements was < 3%.

### Data analyses

Trimmed Spearman-Karber (TSK) procedure was used to calculate the 48-h median lethal concentration (48-h LC50) and the associated 95% confidence interval throughout the study. We used the criterion of ‘non-overlapping 95% confidence intervals’ to determine significant differences (*p* < 0.05) among 48-h LC50 values^35^. Linear regression analyses were performed using SigmaPlot 12.5 (Systat Software Inc., San Jose, CA, USA). We used a nonlinear regression model with the least squares method using SigmaPlot 12.5 (Systat Software Inc., San Jose, CA, USA) to produce the concentration–response curves. Statistical tests for one-way ANOVA followed by Tukey’s post hoc test were performed using Prism 9.1.0 (GraphPad Software, San Diego, CA, USA). The significance level for all statistical analyses was set at *α* = 0.05.

## Supplementary Information

Supplementary Information.

## References

[1] Rand GM (1995). Fundamentals of Aquatic Toxicology: Effects Environmental Fate and Risk Assessment.

[2] Sarma SSS, Nandini S (2006). Review of recent ecotoxicological studies on cladocerans. J. Environ. Sci. Health B.

[3] Beketov MA, Liess M (2012). Ecotoxicology and macroecology—Time for integration. Environ. Pollut..

[4] OECD. *Daphnia* sp., Acute Immobilisation Test and Reproduction Test. No: 202. Organisation for Economic Co-operation and Development, Paris, France (1984).

[5] USEPA. Methods for Measuring the Acute Toxicity of Effluents and Receiving Waters to Freshwater and Marine Organisms. EPA-821-R-02-012. U.S. Environmental Protection Agency, Washington, DC, USA (2002).

[6] Bownik A (2020). Physiological endpoints in daphnid acute toxicity tests. Sci. Total Environ..

[7] Tian-yi C, McNaught DC (1992). Toxicity of methylmercury to *Daphnia pulex*. Bull. Environ. Contam. Toxicol..

[8] Karen DJ (1999). Influence of water quality on silver toxicity to rainbow trout (*Oncorhynchus mykiss*), fathead minnows (*Pimephales promelas*), and water fleas (*Daphnia magna*). Environ. Toxicol. Chem..

[9] Santore RC, Di Toro DM, Paquin PR, Allen HE, Meyer JS (2001). Biotic ligand model of the acute toxicity of metals. 2. Application to acute copper toxicity in freshwater fish and Daphnia. Environ. Toxicol. Chem..

[10] De Schamphelaere KAC, Janssen CR (2002). A biotic ligand model predicting acute copper toxicity for *Daphnia magna*: The effects of calcium, magnesium, sodium, potassium, and pH. Environ. Sci. Technol..

[11] Meyer JS (2005). Toxicity of Dietborne Metals to Aquatic Organisms.

[12] Wang WX (2013). Dietary toxicity of metals in aquatic animals: Recent studies and perspectives. Chin. Sci. Bull..

[13] DeForest DK, Meyer JS (2015). Critical review: Toxicity of dietborne metals to aquatic organisms, critical. Crit. Rev. Environ. Sci. Technol..

[14] Hook SE, Fisher NS (2001). Sublethal effects of silver in zooplankton: Importance of exposure pathways and implications for toxicity testing. Environ. Toxicol. Chem..

[15] De Schamphelaere KAC, Janssen CR (2004). Effects of chronic dietary copper exposure on growth and reproduction of *Daphnia magna*. Environ. Toxicol. Chem..

[16] Besser JM, Brumbaugh WG, Brunson EL, Ingersoll CG (2005). Acute and chronic toxicity of lead in water and diet to the amphipod *Hyalella azteca*. Environ. Toxicol. Chem..

[17] Doke DA, Hudson SL, Dawson JA, Gohlke JM (2014). Effects of early life exposure to methylmercury in *Daphnia pulex* on standard and reduced food ration. Reprod. Toxicol..

[18] Tsui MTK, Wang WX (2007). Biokinetics and tolerance development of toxic metals in *Daphnia magna*. Environ. Toxicol. Chem..

[19] Croteau MN, Luoma SN (2009). Predicting dietborne metal toxicity from metal influxes. Environ. Sci. Technol..

[20] Tsui MTK, Wang WX (2006). Acute toxicity of mercury to *Daphnia magna* under different conditions. Environ. Sci. Technol..

[21] Okamotoa A, Yamamuroa M, Tatarazakoa N (2015). Acute toxicity of 50 metals to *Daphnia magna*. J. Appl. Toxicol..

[22] Meyer JS, Ranville JF, Pontasch M, Gorsuch JW, Adams WJ (2015). Acute toxicity of binary and ternary mixtures of Cd, Cu, and Zn to *Daphnia magna*. Environ. Toxicol. Chem..

[23] USEPA. Short-term Methods for Estimating the Chronic Toxicity of Effluents and Receiving Waters to Freshwater Organisms. EPA-821-R-02-013. U.S. Environmental Protection Agency, Washington, DC, USA (2002).

[24] Tsui MTK, Wang WX (2004). Uptake and elimination routes of inorganic mercury and methylmercury in *Daphnia magna*. Environ. Sci. Technol..

[25] Honeyman BD, Santschi PH (1988). Metals in aquatic systems. Environ. Sci. Technol..

[26] OECD. Guidelines for Testing of Chemicals. *Daphnia magna* Reproduction Test. Organisation for Economic Co-operation and Development, Paris, France (2005).

[27] Monson PL, Brezonik PL (1999). Influence of food, aquatic humus, and alkalinity on methylmercury uptake by *Daphnia magna*. Environ. Toxicol. Chem..

[28] Karimi R, Chen CY, Pickhardt PC, Fisher NS, Folt CL (2007). Stoichiometric controls of mercury dilution by growth. Proc. Natl. Acad. Sci. U.S.A..

[29] Wang WX, Fisher NS (1999). Assimilation efficiencies of chemical contaminants in aquatic invertebrates: A synthesis. Environ. Toxicol. Chem..

[30] McCarty LS, Mackay D (1993). Enhancing ecotoxicological modeling and assessment. Body residues and modes of toxic action. Environ. Sci. Technol..

[31] Luoma SC (1992). Determination of selenium bioavailability to a benthic bivalve from particulate and solute pathways. Environ. Sci. Technol..

[32] Guillard RRL, Smith WL, Chanley MH (1975). Culture of phytoplankton for feeding marine invertebrates. Culture of Marine Invertebrate Animals.

[33] Chow E, Tsui MTK (2019). Elucidating microbial pathways of mercury methylation during litter decomposition. Bull. Environ. Contam. Toxicol..

[34] Ku P (2021). Examination of mercury contamination from a recent coal ash spill into the Dan River, North Carolina, United States. Ecotoxicol. Environ. Saf..

[35] APHA. Standard Methods for the Examination of Water and Wastewaters, 20th ed.; American Public Health Association, Washington, DC, USA (1998).

