# Supplementary material for: Alteration of acute toxicity of inorganic and methyl mercury to Daphnia magna by dietary addition
Source: Sci Rep. 2021 Nov 24;11:22865. doi: 10.1038/s41598-021-02300-4 (PMC8613259; doi:10.1038/s41598-021-02300-4)
Supplement: Supplementary file 1 — Supplementary Information. [file 41598_2021_2300_MOESM1_ESM.docx]

Supplemental Information

**Alteration of acute toxicity of inorganic and methyl mercury to *Daphnia magna* by dietary addition**

Christopher A. Hylton ^a^ and Martin Tsz-Ki Tsui ^a,b,*^

*^a^ Department of Biology, University of North Carolina at Greensboro, Greensboro, NC 27402, USA*

*^b^ School of Life Sciences, The Chinese University of Hong Kong, Shatin, N.T., Hong Kong SAR, China*

*^*^ Corresponding author (M. T.-K. Tsui); address: School of Life Sciences, The Chinese University of Hong Kong, Shatin, N.T., Hong Kong SAR, China; e-mail:* [*mtktsui@cuhk.edu.hk*](mailto:mtktsui@cuhk.edu.hk)

*or* [*tmtsui@uncg.edu*](mailto:tmtsui@uncg.edu)

**Table S1** Measured concentrations in aqueous phase and mobile (surviving) daphnids for the toxicity assays with Hg(II). “-“ means no data due to insufficient immobile daphnids for analysis in that concentration level.

|  | | | **Aqueous phase** | | | **Mobile daphnids** |
| --- | --- | --- | --- | --- | --- | --- |
| **Hg form** | **Treatment** | **Nominal concentration** | **Initial unfiltered (t=0)**  **(µg/L)** | **Final unfiltered**  **(µg/L)** | **Final filtered**  **(µg/L)** | **Biota Hg**  **(mg/kg dry wt.)** |
| Hg(II) | Control | 0.000 | 0.000 | 0.012 | 0.007 | 0.599 |
|  |  | 1.563 | 1.644 | 1.398 | 1.149 | 25.162 |
|  |  | 3.125 | 3.573 | 2.577 | 2.336 | 34.885 |
|  |  | 6.250 | 6.772 | 4.419 | 4.010 | 55.346 |
|  |  | 12.500 | 14.106 | 7.206 | 6.653 | - |
|  |  | 25.000 | 28.485 | 13.304 | 11.478 | - |
| Hg(II) | + Alga | 0.000 | 0.000 | 0.015 | 0.008 | 0.696 |
|  |  | 1.563 | 1.770 | 1.387 | 0.523 | 9.430 |
|  |  | 3.125 | 3.346 | 3.006 | 1.188 | 21.552 |
|  |  | 6.250 | 6.668 | 5.981 | 2.584 | 32.642 |
|  |  | 12.500 | 13.315 | 8.865 | 4.395 | 55.889 |
|  |  | 25.000 | 26.823 | 12.301 | 6.267 | - |
| Hg(II) | + YCT | 0.000 | 0.000 | 0.002 | 0.002 | 0.104 |
|  |  | 1.563 | 1.839 | 0.853 | 0.328 | 8.384 |
|  |  | 3.125 | 3.878 | 2.069 | 0.706 | 13.130 |
|  |  | 6.250 | 6.380 | 3.509 | 0.977 | 24.098 |
|  |  | 12.500 | 12.644 | 6.119 | 1.449 | 26.669 |
|  |  | 25.000 | 25.394 | 11.003 | 1.822 | 65.808 |
| Hg(II) | + Alga & YCT | 0.000 | 0.001 | 0.002 | 0.001 | 0.226 |
|  |  | 1.563 | 1.749 | 0.909 | 0.339 | 9.148 |
|  |  | 3.125 | 3.624 | 1.453 | 0.514 | 16.822 |
|  |  | 6.250 | 6.901 | 3.653 | 0.826 | 28.291 |
|  |  | 12.500 | 13.911 | 5.381 | 1.239 | 40.541 |
|  |  | 25.000 | 26.008 | 8.189 | 2.004 | 81.323 |

**Table S2** Measured concentrations in aqueous phase and mobile (surviving) daphnids for the toxicity assays with MeHg. “-“ means no data due to insufficient immobile daphnids for analysis in that concentration level.

|  | | | **Aqueous phase** | | | **Mobile daphnids** |
| --- | --- | --- | --- | --- | --- | --- |
| **Hg form** | **Treatment** | **Nominal concentration** | **Initial unfiltered**  **(µg/L)** | **Final unfiltered**  **(µg/L)** | **Final filtered**  **(µg/L)** | **Biota Hg**  **(mg/kg dry wt.)** |
| MeHg | Control | 0.000 | 0.000 | 0.011 | 0.000 | 0.106 |
|  |  | 6.250 | 5.086 | 4.830 | 4.629 | 67.627 |
|  |  | 12.500 | 9.680 | 9.141 | 8.914 | 135.569 |
|  |  | 25.000 | 18.826 | 18.188 | 17.413 | 275.635 |
|  |  | 50.000 | 37.732 | 38.026 | 35.416 | - |
|  |  | 100.000 | 74.635 | 73.911 | 73.082 | - |
| MeHg | + Alga | 0.000 | 0.000 | 0.003 | 0.002 | 0.179 |
|  |  | 1.563 | 1.230 | 0.017 | 0.328 | 133.357 |
|  |  | 3.125 | 2.437 | 1.637 | 1.090 | 180.397 |
|  |  | 6.250 | 4.947 | 3.914 | 1.375 | 175.581 |
|  |  | 12.500 | 9.959 | 8.984 | 5.464 | 178.360 |
|  |  | 25.000 | 19.882 | 17.965 | 14.357 | - |
| MeHg | + YCT | 0.000 | 0.001 | 0.003 | 0.001 | 0.175 |
|  |  | 6.250 | 5.577 | 1.607 | 0.199 | 201.721 |
|  |  | 12.500 | 12.655 | 4.840 | 1.207 | 316.616 |
|  |  | 25.000 | 23.715 | 12.875 | 3.242 | 326.254 |
|  |  | 50.000 | 48.480 | 35.283 | 23.059 | - |
|  |  | 100.000 | 92.943 | 79.067 | 57.050 | - |
| MeHg | + Alga & YCT | 0.000 | 0.001 | 0.003 | 0.001 | 0.305 |
|  |  | 6.250 | 6.929 | 2.177 | 0.338 | 193.719 |
|  |  | 12.500 | 10.861 | 3.902 | 0.728 | 297.498 |
|  |  | 25.000 | 22.072 | 12.141 | 2.834 | 265.115 |
|  |  | 50.000 | 43.814 | 29.441 | 12.647 | - |
|  |  | 100.000 | 87.786 | 47.088 | 4.161 | - |
